# miR-21: an oncomir on strike in prostate cancer

**DOI:** 10.1186/1476-4598-9-12

**Published:** 2010-01-21

**Authors:** Marco Folini, Paolo Gandellini, Nicole Longoni, Valentina Profumo, Maurizio Callari, Marzia Pennati, Maurizio Colecchia, Rosanna Supino, Silvia Veneroni, Roberto Salvioni, Riccardo Valdagni, Maria Grazia Daidone, Nadia Zaffaroni

**Affiliations:** 1 Department of Experimental Oncology and Molecular Medicine, Fondazione IRCCS Istituto Nazionale dei Tumori, Via Venezian, 1, Milan, 20133, Italy; 2 Department of Pathology, Fondazione IRCCS Istituto Nazionale dei Tumori, Via Venezian, 1, Milan, 20133, Italy; 3 Department of Urology, Fondazione IRCCS Istituto Nazionale dei Tumori, Via Venezian, 1, Milan, 20133, Italy; 4 Prostate Program, Scientific Directorate, Fondazione IRCCS Istituto Nazionale dei Tumori, Via Venezian, 1, Milan, 20133, Italy

## Abstract

**Background:**

Aberrant expression of microRNAs, small non-coding RNA molecules that post-transcriptionally repress gene expression, seems to be causatively linked to the pathogenesis of cancer. In this context, miR-21 was found to be overexpressed in different human cancers (e.g. glioblastoma, breast cancer). In addition, it is thought to be endowed with oncogenic properties due to its ability to negatively modulate the expression of tumor-suppressor genes (e.g. *PTEN*) and to cause the reversion of malignant phenotype when knocked- down in several tumor models. On the basis of these findings, miR-21 has been proposed as a widely exploitable cancer-related target. However, scanty information is available concerning the relevance of miR-21 for prostate cancer. In the present study, we investigated the role of miR-21 and its potential as a therapeutic target in two prostate cancer cell lines, characterized by different miR-21 expression levels and *PTEN *gene status.

**Results:**

We provide evidence that miR-21 knockdown in prostate cancer cells is not sufficient *per se *i) to affect the proliferative and invasive potential or the chemo- and radiosensitivity profiles or ii) to modulate the expression of the tumor-suppressors PTEN and Pdcd4, which in other tumor types were found to be regulated by miR-21. We also show that miR-21 is not differently expressed in carcinomas and matched normal tissues obtained from 36 untreated prostate cancer patients subjected to radical prostatectomy.

**Conclusions:**

Overall, our data suggest that miR-21 is not a central player in the onset of prostate cancer and that its single hitting is not a valuable therapeutic strategy in the disease. This supports the notion that the oncogenic properties of miR-21 could be cell and tissue dependent and that the potential role of a given miRNA as a therapeutic target should be contextualized with respect to the disease.

## Background

MicroRNAs (miRNAs) are small non-coding RNA molecules that regulate gene expression by influencing the stability or the translational efficiency of target mRNAs [1]. Their tissue- and time-dependent expression influences protein production during distinct cellular processes [1], and their aberrant expression is causative in the pathogenesis of several diseases, including cancer [2-4]. Many miRNAs have been identified as crucial players in different human tumors [3].

miR-21 has high expression levels in glioblastoma [5], breast cancer [6,7] and tumors of the gastrointestinal tract [8-14] compared to normal tissues. In addition, it has been reported to counteract the expression of putative tumor-suppressive targets, such as phosphatase and tensin homolog deleted on chromosome 10 (PTEN), programmed cell death 4 (Pdcd4), tropomyosin 1, maspin and reversion-inducing cysteine-rich protein with kazal motifs [7,10-12,15-21]. On the basis of these findings, miR-21 has been proposed to play a pivotal role in the onset of several tumor types. Accordingly, its antisense-mediated knockdown has been reported to impair the growth, to induce apoptosis and to reduce the migration and invasion of cancer cells highly expressing miR-21 [5,10,20-27]. Altered miR-21 expression levels have been also reported to affect the sensitivity to different anticancer agents of cholangiocarcinoma and pancreatic, non-small cell lung, glioma and ovarian cancer cells [11,28,29]. On the basis of these findings, miR-21 has been referred to as an "oncomir" (i.e., a miRNA with oncogenic properties), and the possibility to negatively interfere with its expression or with its interaction with downstream targets has been suggested as a potential anticancer therapeutic approach.

Scanty information is available concerning the relevance of miR-21 for prostate cancer (PCa). In this study, we investigated the effects of miR-21 down-regulation in PCa cell lines expressing it at high levels and characterized by a different status of PTEN, and provided evidence that miR-21 knockdown is not sufficient *per se *to significantly modify the proliferative potential and the chemo- and radiosensitivity profiles of PCa cells. Our findings suggest that the single hitting of miR-21 would not be a valuable therapeutic strategy in this disease. The hypothesis that miR-21 is not a major player in PCa is also corroborated by the evidence that miR-21 is not differently expressed in prostate carcinomas and matched normal tissues obtained from 36 untreated patients subjected to radical prostatectomy.

Our data support the notion that the oncogenic properties of miR-21 would be cell- and tissue-dependent and that the potential role of a given miRNA as a therapeutic target should be contextualized with respect to the disease.

## Results and discussion

**Figure 1:**
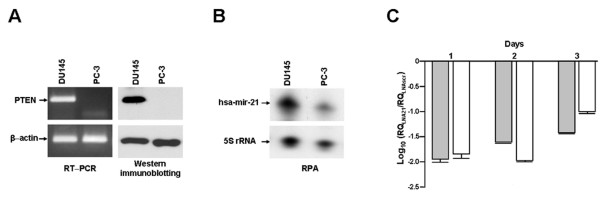
**Characterization of PCa cells for miR-21 and PTEN expression**. (A) RT-PCR and western immunoblotting, and (B) RNase protection assay showing the basal expression levels of PTEN and miR-21 in DU145 and PC-3 cell lines. (C) qRT-PCR showing the down-modulation of free miR-21 in DU145 (grey bars) and PC-3 cells (white bars) exposed to LNA21. Data are reported as relative quantity (RQ) of LNA21- over LNAScr-treated cells and represent mean values ± SD of at least three independent determinations.

**Figure 2:**
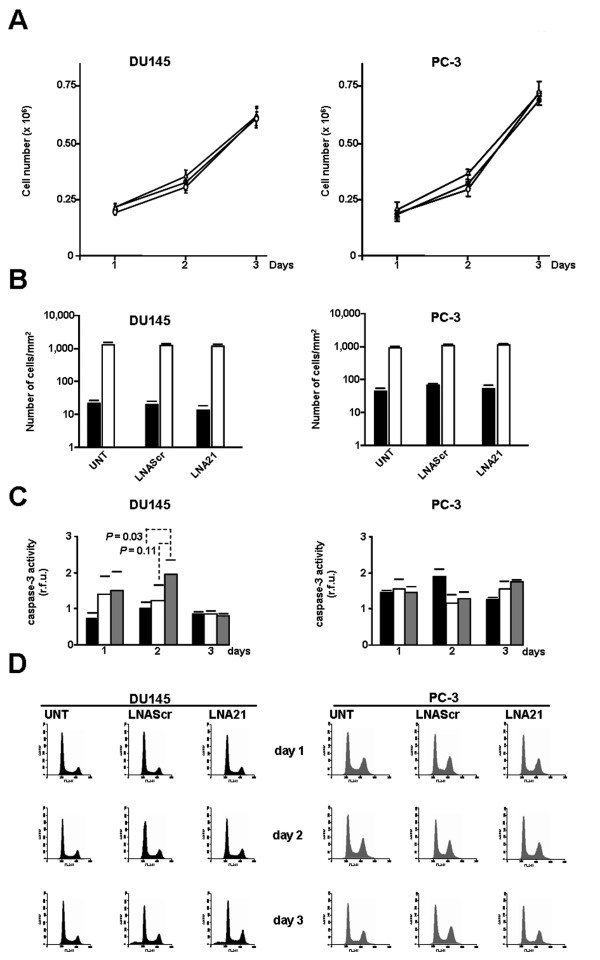
**Analysis of the effects of miR-21 knockdown on PCa cell behavior**. (A) Growth curves of untreated (white triangle), LNAScr (black circle)- or LNA21 (white circle)- transfected DU145 and PC-3 cells. (B) Analysis of the migrating (black bars) and invading (white bars) capabilities of PCa cells (number of cells/mm^2^) at day 3, in untreated (UNT) and LNAScr- or LNA21-transfected cells. (C) Time-course determination of caspase-3 catalytic activity in untreated (black bars) and LNAScr- (white bars) or LNA21-transfected (grey bars) cells. R.f.u.: relative fluorescence units. (D) Time-course analysis of the cell cycle in UNT or oligomer-transfected PCa cells. Data represent mean values ± SD of at least three independent experiments.

Several studies have demonstrated that miR-21 is an oncomir with anti-proliferative and anti-apoptotic functions [30]. In several cancer cell lines highly expressing miR-21, its down-regulation by antisense oligomers resulted in growth suppression, induction of apoptosis and impairment of migration and invasion [5,10,20-27]. Scanty information has been obtained thus far regarding whether or not miR-21 is involved in PCa. Through a loss-of-function approach, we functionally investigated the role of miR-21 and its potential as a therapeutic target in two experimental models of androgen-independent PCa, DU145 and PC-3 cells. The two cell lines are characterized by a different *PTEN *status (Figure 1A) and distinct expression levels of miR-21 (Figure 1B). A locked nucleic acid (LNA)-modified anti-miR-21 oligomer (LNA21) was used to interfere with miR-21 function. The exposure of PCa cells to a carboxyfluorescein (FAM)-labeled LNA21 resulted in a transfection efficiency of almost 100%, as assessed by flow cytometry 24 h after transfection (Additional file 1, Figure S1). Administration of LNA21 resulted in a nearly complete reduction of free mature miR-21 abundance (Figure 1C), as revealed by quantative reverse transcriptase-polymerase chain reaction (qRT-PCR) and northern blotting (Additional file 1, Supplementary Methods and Figure S2). Such an effect was appreciable starting from day 1 after a 4-h transfection with the oligomer (quantification of free miR-21 levels: 1.13 ± 0.1% and 1.42 ± 0.25% compared to DU145 and PC-3 cells transfected with a scrambled oligomer (LNAScr), respectively) and still present at day 3 (3.80 ± 0.13% and 10.1 ± 0.85%) (Figure 1C). The impairment of miR-21 function -- due to either the degradation or the reduction of free miR-21 as a consequence of LNA21-mediated "decoy effect" -- failed to affect the growth of PCa cells. In fact, the growth curves of LNA21 transfectants were superimposable to those of untreated or LNAScr-treated cells (Figure 2A). In addition, when compared to untreated or LNAScr-transfected cells, no changes in the number of migrating cells were observed in DU145 or PC-3 cells transfected with LNA21, as assessed by the Transwell assay at day 3 after transfection (Figure 2B). Again, when the assay was performed in the presence of matrigel, as a surrogate of the extracellular matrix, we could not detect any impairment of the invading capabilities of PCa cells upon interference with miR-21 function (Figure 2B). In contrast with our data, Li *et al*. [31] recently showed that a 2'-*O*-methyl-RNA oligomer against miR-21 significantly reduced the migration and invasion capabilities of DU145 and PC-3 cells. Such a discrepancy could reside in the different chemistry of the anti-miR-21 oligomers used. It has been demonstrated that the 2'-sugar and backbone modifications significantly affect the ability of antisense oligomers to interfere with the function and activity of their targets. Specifically, it was found that at concentrations higher than 30 nM some anti-miR-21-modified oligomers, primarily 2'-*O*-methyl-phosphorothioates, had negative effects on cell behavior compared to LNA oligomers [32-34], resulting in a significant inhibition of cell proliferation independently of target down-regulation. In this context, Li *et al*. [31] did not evaluate the extent of miR-21 down-regulation in their study. It has also been reported that the same interactions influencing the tolerability of the chemical modification of small interfering RNA (siRNA) passenger strands hold true for modified anti-miRNA oligomers. Specifically, using a model siRNA based on miR-21 sequence, a set of modified passenger strands were paired to an unmodified guide strand RNA and tested for their ability to reduce the mRNA levels of PTEN -- a miR-21 target -- in HeLa cells [32]. It has been found that 2'-*O*-methyl-RNA oligomers, with either phosphodiester or phosphorothioate backbones, were well tolerated as a PTEN siRNA passenger strand, paradoxically triggering the activation of the RNA interference pathway, and that their activity as siRNA was inversely correlated with their anti-miR-21 function [32]. When we administered a 2'-*O-*methyl-modified anti-miR-21 oligomer to DU145 cells, a ~50% inhibition of cell growth was observed in spite of a less efficient down-regulation of miR-21 (10^-1^-fold) compared to that obtained using the LNA21 (10^-2^-fold) (Additional file 1, Figure S3), suggesting the latter as a more specific tool to investigate miR-21 function.

In our hands, miR-21 knockdown did not trigger apoptosis as revealed by the lack of substantial caspase-3 activation (Figure 2C) in either cell line. However, a ~1.5-2-fold increase in caspase-3 catalytic activity was appreciable in DU145 cells at day 2 after the transfection with LNA21 (Figure 2C), compared to untreated (*P *= 0.03) or LNAScr-transfected cells (*P *= 0.11), which disappeared one day later despite the persistent impairment of miR-21 (Figure 2C). Such a temporary increase in caspase-3 activity was likely insufficient to overcome the threshold for the induction of apoptosis. In fact, we observed a low percentage (<5% of the overall cell population) of cells with an apoptotic nuclear morphology after propidium iodide staining (data not shown). In addition, we failed to detect a sub-G_0/1 _peak on DNA plots at all time points considered (Figure 2D), except for a pelting sub-G_0/1 _peak in DU145 cells at day 3 after the transfection with either LNA21 or LNAScr (Figure 2D). These data would indicate that such a small fraction of apoptotic cells reflects a stress response of cells to treatment rather than being specifically related to miR-21 depletion.

In some tumor models other than PCa, the overexpression of miR-21 has been associated with chemoresistance, and the modulation of its expression levels has been proven to affect the activity of anticancer agents [11,28,29]. Specifically, it has been shown that the overexpression of miR-21 increased the resistance to gemcitabine of PANC-1 and SUIT-2 pancreatic cancer cells [28] as well as of Mz-ChA-1 cholangiocarcinoma cells [11], through the activation of PI-3 kinase and AKT/mTOR signaling as a consequence of the suppression of PTEN by miR-21 [11]. In addition, an increase in topotecan-induced growth inhibition was appreciable in MCF-7 breast cancer cells pre-incubated with an anti-miR-21 oligonucleotide [29].

**Figure 3:**
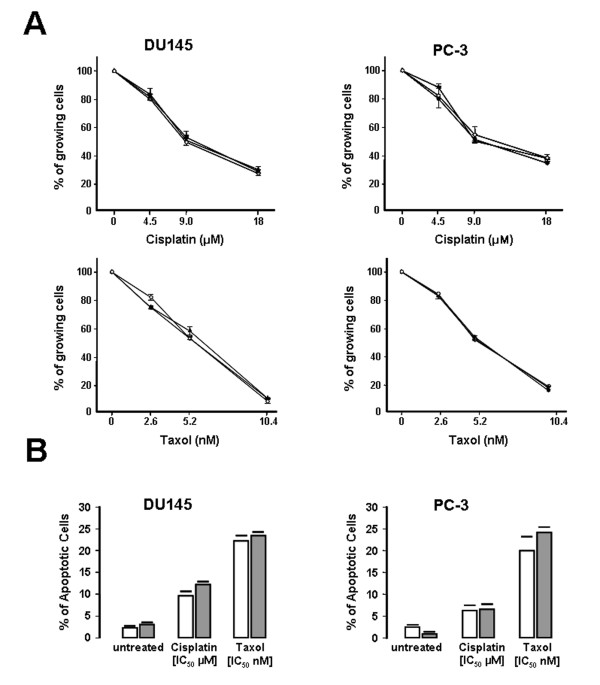
**Analysis of miR-21 knockdown on the chemosensitivity profiles of PCa cells**. (A) Growth inhibition curves of untreated (black triangle), LNAScr (black circle)- or LNA21 (white circle)-transfected PCa cells exposed to increasing concentrations of cisplatin or taxol. Data are reported as percentage of growing cells compared to untreated controls and represent mean values ± SD of at least three independent experiments. (B) Quantification of cells with an apoptotic nuclear morphology by propidium iodide staining of PCa cells transfected with LNAScr (white bars) or LNA21 (grey bars) and exposed to IC_50 _of cisplatin or taxol. Data are reported as the average (± SD) of the percentage of apoptotic cells in the overall cell population.

Based on these findings, we investigated whether miR-21 down-regulation could be exploited as a strategy to sensitize PCa cells to treatment with anticancer agents, often inefficient for the clinical therapy of PCa [35]. LNA21-transfected DU145 cells were exposed to anticancer drugs with different mechanisms of action (i.e., cisplatin and taxol) and ionizing radiation. Results indicated that the impairment of miR-21 function did not modify the chemosensitivity of DU145 or PC-3 cells, since the growth curves of LNA21- transfected cells were superimposable to those of untreated or LNAScr- transfected cells, as evaluated at day 3 after the exposure to cisplatin or taxol (Figure 3A). Accordingly, no differences in drug-induced apoptosis, evaluated as the percentage of propidium iodide-stained cells, were appreciable in LNA21-transfected cells compared to cells exposed to LNAScr (Figure 3B).

A recent study reported the effects of manipulating miR-21 levels on drug resistance of cancer cell lines characterized by different miR-21 basal expression levels [29]. Upon ectopic expression of miR-21, an increased resistance to doxorubicin was observed in A549 non-small cell lung cancer and OVCAR3 ovarian cancer cells, whereas an increased sensitivity to the same drug was observed in SNB19 glioma cells [29]. Following miR-21 knockdown, all three cancer cell lines showed increased sensitivity to topotecan, whereas manipulating the expression levels of miR-21 did not affect their sensitivity to 10-hydroxycamptothecin [29]. These observations suggest that the effects arising from interference with the expression of a specific miRNA could be dependent on the cell model.

**Figure 4:**
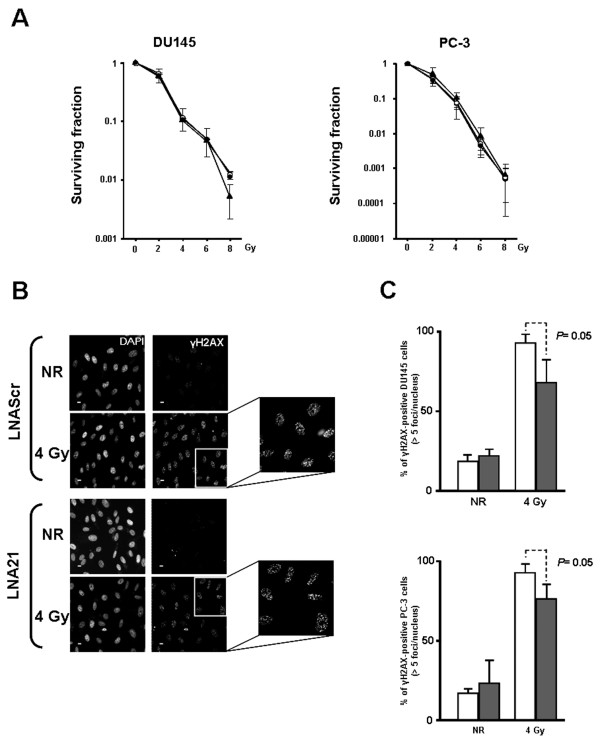
**Analysis of miR-21 knockdown on the radiosensitivity profiles of PCa cells**. (A) Clonogenic survival curves of untreated (black triangle), LNAScr (black circle)- or LNA21 (white circle)-transfected cells calculated on day 12 after exposure to increasing doses (2-8 Gy) of γ- radiation. Data shown on the plots represent the inter-experiment averages (± SD) calculated from at least three intra-experiment averages. (B) Representative immunofluorescence analysis of γ- H2AX induction in LNAScr- or LNA21-treated DU145 cells exposed to γ- radiation (4 Gy). Nuclei were counterstained with 4',6-diamidino-2-phenylindole. Scale bar: 10 μm. Magnification: × 40. NR, no radiation. (C) Quantification of γ- H2AX foci in DU145 (*top panel*) and PC-3 cells (*bottom panel*). Data are reported as percentage of γ- H2AX-positive cells in the overall cell population (mean values ± SD).

No variation in the radiosensitivity profile of miR-21-knocked-down PCa cells was observed on clonogenic cell survival curves generated after the exposure to γ-radiation (2-8 Gy) with respect to those of controls (Figure 4A). However, when cultures were scored for the presence of γ-H2AX foci, a slight but significant decrease (*P *= 0.05) in the percentage of cells harboring DNA damage (>5 γ-H2AX foci/nucleus) was appreciable in miR-21-depleted cells (Figure 4B, C). This finding would suggest that reducing miR-21 expression levels could, at least in part, protect PCa cells from radiation by limiting the induction of DNA damage, as already described for other miRNAs in distinct cell systems. Specifically, it has been shown that the *in vitro *down-regulation of *let-7 *family members (i.e., *let-7a *and *let-7b*) resulted in an increased radioresistance of A549 lung cancer cells [36]. Similarly, LNCaP cells knocked-down for miR-521 showed a reduced sensitivity to radiation, as a consequence of the up-regulation of Cockayne syndrome protein A, a DNA repair factor targeted by miR-521 [37].

**Figure 5:**
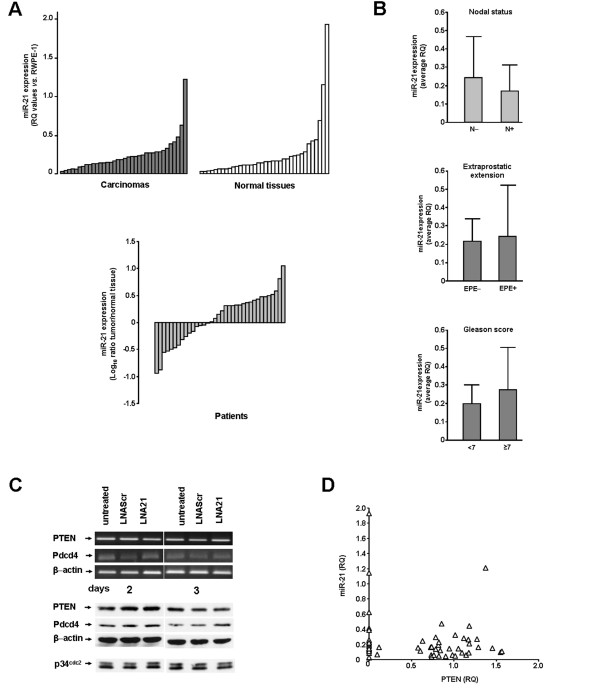
**miR-21 expression in carcinomas and normal prostate tissues**. (A) Quantification of miR-21 expression levels (*top panel*) in carcinomas and matched normal tissues obtained from 36 patients subjected to radical prostatectomy. Data are reported as RQ of miR-21 expression with respect to an internal calibrator (RWPE-1). Analysis of miR-21 expression (*bottom panel*) reported as Log_10 _of the ratio tumor/matched normal tissue for each patient (RQ_tumor_/RQ_normal_). (B) miR-21 expression levels (average RQ ± SD) as a function of nodal status, extraprostatic extension (EPE) of the disease and Gleason score. (C) Representative RT-PCR and western immunoblotting showing the expression of miR-21 validated target genes in untreated and LNAScr- or LNA21-treated DU145 cells, at days 2 and 3 after transfection. (D) Scatterplot showing the lack of a correlation between RQ expression values of miR-21 and PTEN mRNA in clinical specimens.

Overall, our data suggest that miR-21 is not a major player in PCa, as its single hitting is not enough to counteract the proliferative potential of PCa cells or to affect their sensitivity to anticancer drugs and radiation. In addition, normal prostate RWPE-1 cells - which express high levels of miR-21 (Additional file 1, Figure S4) - did not show any biological response upon either the depletion or up-modulation of the miRNA (Additional file 1, Figure S5). The negligible role of miR-21 in PCa is also supported by expression data in experimental and clinical prostate models. Specifically, qRT-PCR analysis revealed that androgen-dependent PCa cells (LNCaP and VCaP) express markedly lower levels of miR-21 than androgen-independent DU145 and PC-3 cells and that normal prostate RWPE-1 cells are characterized by amounts of miR-21 similar to those of DU145 cells (Additional file 1, Figure S4). As regards the clinical setting, we found that overall miR-21 expression levels were similar in tumor and nonneoplastic tissue specimens (average RQ 0.23 ± 0.21 and 0.24 ± 0.35, respectively;*P *= 0.86) obtained from 36 untreated patients subjected to radical prostatectomy (Figure 5A, *top*). In addition, miR-21 was not consistently up-regulated in carcinomas compared to the matched normal tissues from each patient (Figure 5A, *bottom*).

No significant differences in miR-21 expression were observed as a function of nodal status (average RQ 0.243 ± 0.223 and 0.168 ± 0.143 in localized (N-) *vs*. local-regionally disseminated (N+) disease; *P *= 0.35), extraprostatic extension of the disease (0.219 ± 0.128 and 0.245 ± 0.272 in negative *vs*. positive specimens; *P *= 0.730), or Gleason score (0.200 ± 0.101 and 0.274 ± 0.272 in specimens scored <7 *vs*. ≥ 7; *P *= 0.45) (Figure 5B).

miR-21 was proposed to be one of the six miRNAs whose overexpression represents the signature for solid tumors [38]. However, survey of available data on miRNA expression in PCa clinical specimens showed it was not differently expressed in tumor and normal prostate tissues [39-42] or in localized and metastatic PCa [43] in 5 out of 6 studies [38-43] (Table 1 and Additional file 1, Supplementary Methods). This suggests that miR-21 up- modulation is not a common or relevant event in prostate tumorigenesis.

**Table 1:** miR-21 expression in human prostate^1^

Study	miR-21 expression^2^	Case series	Analytical method
Volinia *et al*. [38]	↑	56 PCa; 7 normal prostate tissues from non-cancer individuals	In-house microchip oligonucleotide microarray
Lu *et al*. [39]	=	6 PCa; 8 normal prostate tissues	Bead-based flow cytometry detection system
Porkka *et al*. [40]	=	9 PCa; 4 benign prostatic hyperplasias	In-house oligonucleotide microarray
Ozen *et al*. [41]	=	16 PCa; 10 benign prostatic hyperplasias	miRvana miRNA bioarrays
Ambs *et al*. [42]	=	60 macrodissected PCa; 16 surrounding non-tumor prostate tissues	microRNA microarray (Ohio State University Comprehensive Cancer Center, Version 3.0)
Leite *et al*. [43]	=	18 localized high-grade PCa from radical prostatectomies; 4 metastatic, androgen-independent PCa	TaqMan miRNA qRT-PCR assay

^1^The analysis was carried out as reported in Additional file 1, Supplementary Methods.

^2^Up-modulation (↑) or no differential expression (=) of miR-21 in prostate tumors *vs*. control tissue used in the study (i.e., normal prostate or benign prostatic hyperplasia or localized high-grade PCa).

The peculiar aspect that makes a miRNA a sophisticated engine to finely tune the expression of genes resides in its possibility to simultaneously target several cellular factors [2,3]. Consequently, the function of a given miRNA strictly depends on the cell phenotype and, therefore, on the presence of its targets in the tissue where it is expressed. In this context, we wondered whether the absence of any evident effect on miR-21 knockdown could rely on the lack of its key downstream targets or in a reduced ability to suppress them in PCa cells. DU145 cells inherently express PTEN (Figure 1A), a direct target of miR-21 as predicted by distinct target prediction analysis softwares (e.g., miRanda and RNAhybrid) and validated by functional analyses [7,11,15]. Our data showed that upon exposure to LNA21, the expression levels of PTEN (mRNA and protein) were not substantially altered, compared to untreated or LNAScr-transfected cells (Figure 5C). This finding suggests that miR-21 is not involved in the regulation of PTEN expression in DU145 cells.

To verify such a hypothesis, we wondered whether in a clinical setting there was an inverse correlation between miR-21 expression and that of PTEN, as would be expected in the presence of an intimately related miRNA and target gene pair. The analysis of PTEN and miR-21 expression carried out in a subset (n = 29) of clinical specimens, for which residual RNA was available, did not show any significant correlation (r_S _= -0.00717; *P *= 0.957) in the expression levels of the two factors (Figure 5D). It cannot be excluded that the miR-21 responsive elements in the 3'-untranslated region (UTR) of PTEN could be inaccessible in PCa cells as a consequence of folding constraints or mutations that disable the interaction with miR-21, thus enabling the gene to evade regulation by the miRNA.

It is noteworthy that the number and nature of specific miRNA target sequences within the 3'-UTR of mRNAs can significantly affect the sensitivity of a target to the action of a related miRNA. In this context, it has been reported that single nucleotide polymorphisms in miRNA binding sites, which hamper the miRNA-mediated regulation of gene expression, can be associated to cancer predisposition [44]. Specifically, a variant allele at a single nucleotide polymorphism in a *let-7 *complementary site in the 3'-UTR of *k-ras *was found to be associated with a 1.4- to 2.3- fold increased risk for non-small-cell lung cancer among moderate smokers [44]. To our knowledge, the occurrence of single nucleotide polymorphisms in the 3'-UTR of miR-21 targets has not been described thus far in PCa cells.

Consistent with the inverse relationship between a miRNA and its target gene, a slight up-modulation of Pdcd4 protein -- a validated target of miR-21 in different cancer experimental models [12,16-19,27] -- was appreciable 3 days after the exposure of DU145 cells to LNA21 compared to untreated or LNAScr-transfected cells (Figure 5C). However, Pdcd4 downstream signaling pathway [45] was not substantially impaired upon miR-21 down-modulation, as indicated by the comparable levels of p34^cdc2 ^in the different cultures (Figure 5C), which was reflected by the lack of cell cycle perturbations (Figure 2D). Similarly, no change in the expression of Pdcd4 or p34^cdc2 ^was appreciable in the PTEN-negative PC-3 cells upon miR-21 depletion (Additional file 1, Figure S6), although in these cells we failed to properly detect Pdcd4 protein, perhaps due to the fact that its expression levels are below the threshold for detection by western blotting. These findings were also corroborated by the luciferase gene reporter assay, in which a slight but not statistically significant down-modulation of luciferase activity was observed in DU145 and PC-3 cells co-transfected with Luc-Pdcd4WT plasmid (harboring the 3'-UTR of Pdcd4 [27]) and a synthetic precursor of miR-21, compared to a control oligomer (Additional file 1, Figure S7). Moreover, no perturbations of luciferase activity were observed in cells exposed to either LNA21 or LNAScr (Additional file 1, Figure S7).

Overall, our findings indicate that the persistent and marked depletion of miR-21 in PCa cells does not result in an efficient modulation of the downstream factors that, in other tumor histotypes, have been associated to its oncogenic functions, thus explaining the reason for its failure to counteract PCa. Such a scenario could depend on the fact that those factors (e.g., PTEN or Pdcd4) undergo multiple hitting by distinct miRNAs, making their expression levels dependent on the miRNAs "dowry" of a cell. In addition, an imbalance in favor of the deregulated expression of other pro-survival and anti-apoptotic miRNAs could be responsible for the ability of PCa cells to overcome the antitumor phenotype that in other tumor experimental models arises following miR-21 knockdown [5,10,20-27]. In this context, it would be useful to investigate whether the simultaneous down-regulation of miR-21 and other still unidentified miRNAs, converging on miR-21 target genes, synergizes in counteracting the proliferative potential of PCa cells. Such a strategy could represent a suitable multi-target therapeutic approach to achieve spontaneous or treatment-induced PCa cell death.

## Conclusions

We have provided compelling evidence that miR-21 is not *per se *a central player in the onset of PCa and that its single hitting does not represent a valuable therapeutic intervention in such a disease. Our findings contribute to support the notion that the oncogenic properties of miR-21 - and generally speaking that of any miRNA- could be cell and tissue dependent and that its potential role as a biomarker or therapeutic target should be put in the context of a given disease.

On the basis of available data, it is clear that we are still far from the precise identification of miRNAs potentially relevant for the initiation and progression of PCa. Even if further investigations are warranted to unequivocally identify PCa-associated miRNAs, there is evidence that deregulated expression of other miRNAs, instead of miR-21, could represent a prominent event responsible for the onset of the disease [46-48]. Such evidence puts in the limelight the opportunity to successfully exploit these "alternative" miRNAs as new biomarkers for diagnosis or prognostication as well as suitable tools or targets for future therapeutic interventions in PCa.

## Methods

### Experimental models

Human PCa cell lines (DU145 and PC-3) were obtained from American Type Culture Collection (Rockville MD). Cells were resuscitated soon after arrival, cultured in RPMI 1640 medium with 10% fetal calf serum and maintained in 5% CO_2 _at 37°C in separate incubators. Carcinoma and matched normal prostate tissues were obtained, with appropriate informed consent and Institutional Review Board approval, from 36 untreated PCa patients subjected to radical prostatectomy. Freshly frozen surgical blocks, stored in the Institutional Frozen Tumor Bank, were carefully dissected by the pathologist using hematoxylin-eosin-stained sections as a template to identify areas containing at least 70% of tumor or normal cells.

### miRNA and gene expression analysis

Total RNA was isolated from cell cultures and clinical samples through Trizol reagent (Invitrogen, San Giuliano Milanese, Italy) as previously described [49]. miR-21 expression levels were evaluated by RNase protection assay as described in Additional file 1, Supplementary Methods and Table S1. For gene expression studies, cDNA was randomly primed from 0.5 μg RNA and amplified using the GeneAmp RNA PCR Core kit (Applied Biosystems, Monza, Italy). β-actin was used as PCR internal control.

Quantification of mature miR-21 and mRNA expression levels was assessed by qRT-PCR using specific TaqMan^® ^Assays (Applied Biosystems). Amplifications were run on the 7900HT Fast Real-Time PCR System. Data were analyzed by SDS 2.2.2 software (Applied Biosystems) and reported as relative quantity (RQ) with respect to a calibrator sample using the 2^-ΔΔCt ^method. U6 snRNA and RNaseP were used as normalizers.

All primer sets used are listed in Additional file 1, Tables S2-S3.

### Cell-based experiments

Cells seeded at the appropriate density were transfected for 4 h at 37°C with 100 nM anti-miR-21 (LNA21) or scramble (LNAScr) miRCURY™ knockdown probes (Exiqon, Vedbaek, Denmark) using Lipofectamine2000™ (Invitrogen), according to the manufacturer's instructions. Cell growth was evaluated at days 1-3 after transfection by counting cells in a particle counter (Coulter Counter, Beckman Coulter, Fullerton, CA).

For the migration assay, cells were transferred to the upper chamber (2 × 10^5 ^cells/well) of 24-well Transwell plates (Costar, Corning Incorporated, Corning, NY) in serum-free medium and chemoattractant (i.e., conditioned medium obtained by incubating growing cells in a medium without serum for 24 h) was added to the lower chamber. After a 5-h incubation at 37°C, cells in the upper side were cleaned off and filters were fixed in 99% ethanol and stained with a solution of 0.4% sulforhodamine B in 1% acetic acid. Migrated cells were counted under a microscope. The same procedure was used for invasion assay, except that cells were seeded at 3 × 10^5 ^cells/well, Transwell chambers coated with 12.5 μg of Matrigel/well (BD Biosciences, San Jose, CA), and samples processed after a 24-h incubation.

Caspase-3 catalytic activity was measured using the APOPCYTO/caspase-3 assay kit (MBL International, Naka-ku Nagoya, Japan). Total protein extracts and the specific fluorogenic substrate *N*-acetyl-Asp-Glu-Val-Asp-pNA (DEVD-pNA) were mixed and incubated for 1 h at 37°C, and the hydrolysis of DEVD-pNA was monitored by spectrofluorometry at λ460 nm. The presence of a sub-G_1 _peak suggestive of apoptosis [50] was evaluated by a FACScan flow cytometer (Becton Dickinson, Franklin Lake, NJ) on cells fixed in pre-cooled 70% ethanol and stained with a solution containing 50 μg/ml propidium iodide, 50 mg/ml RNase and 0.05% Nonidet P40 for 30 min at 4°C. A minimum of 3 × 10^4 ^events was measured for each sample, and the sub-G_1 _peak was detected on DNA plots by CellQuest software according to the Modfit model (Becton Dickinson). An aliquot of propidium iodide-stained cells was spotted onto glass slides and examined under a fluorescence microscope for the presence of nuclei with an apoptotic morphology. The percentage of apoptotic cells was determined by scoring at least 500 cells for each sample.

For the evaluation of chemo- and radiosensitivity, cell growth analysis and clonogenic assay were performed, respectively. For the chemosensitivity assay, the growth of LNA-transfected PCa cells was evaluated by cell counting at day 3 after a 1-h or 24-h exposure to increasing concentrations of cisplatin (0-18 μM) or taxol (0-10 nM), respectively. For irradiation experiments, one day after exposure to LNAs, exponentially growing cells were harvested and irradiated (2-8 Gy) at room temperature using a ^137^Cs γ irradiator (IBL-437) at a dose rate of 7.2 Gy per min, plated at appropriate concentrations in plastic dishes, and incubated at 37°C for 12 days. Colonies were stained with crystal violet in 70% ethanol and counted under the microscope. The colony-forming efficiency was calculated from the number of colonies (consisting of at least 50 cells) counted and the number of morphologically intact single cells seeded, and the surviving fractions of treated cells relative to non-irradiated cells were determined.

### Immunoblotting and immunofluorescence analyses

For immunoblotting, proteins were fractioned by SDS-PAGE and transferred onto Hybond nitrocellulose membranes (GE Healthcare, Amersham, UK). Filters were blocked in PBS-Tween-20 in 5% skim milk and probed with antibodies raised against PTEN (Abcam, Cambridge, UK), Pdcd4 (Abcam), and p34^cdc2 ^(Santa Cruz Biotechnology, Santa Cruz, CA), which were visualized by a SuperSignal^® ^West PICO chemiluminescent detection system (Thermo Scientific, Rockford, IL). β-actin was used as equal protein loading control.

For immunofluorescence analyses, cells grown on glass coverslips were fixed with 4% formaldehyde and probed with primary anti-γ-H2AX (Abcam) and secondary AlexaFluor594 (Invitrogen) antibodies. Images were acquired by a Nikon Eclipse E600 microscope using ACT-1 software (Nikon Corporation, Japan) and processed with Adobe Photoshop Image Reader 7.0.

### Statistical analyses

Two-sided Student's *t *test was used to analyze the differences in miR-21 expression levels, cell growth, migration, invasion, caspase-3 activity and chemo- and radiosensitivity profiles. Spearman's correlation coefficient (r_S_) with associated *P *value was calculated for PTEN mRNA and miR-21 expression in clinical samples. Two-tailed *P *values < 0.05 were considered statistically significant.

## Competing interests

The authors declare that they have no competing interests.

## Authors' contributions

MF conceived the study, performed cell-based experiments and the chemo- and radiosensitivity assays, collected and analyzed data, and wrote the paper. PG participated in the design of the study, performed cell-based experiments, RT-PCR and qRT-PCR analyses and contributed in manuscript preparation. NL carried out immunoblotting and immunofluorescence analyses. VP performed Northern blotting and luciferase reporter assays. MC analyzed publicly available microarray data. MP acquired funding and assessed caspase catalytic activity. MC dissected clinical samples making them eligible for molecular analyses. RS performed migration and invasion assays. SV collected informed consent from patients and managed the data base of clinical and pathological information. RS selected patients eligible for being included in the study and provided surgical specimens. RV acquired funding and critically revised the manuscript. MGD performed statistical analyses and critically revised the manuscript. NZ acquired funding, supervised the experiments, analyzed data and contributed in manuscript preparation. All authors read and approved the final manuscript.

## Supplementary Material

Additional file 1 **Supplementary Information**. Supplementary methods, tables and figures. Click here for file
