# Supplementary material for: miR-21: an oncomir on strike in prostate cancer
Source: Mol Cancer. 2010 Jan 21;9:12. doi: 10.1186/1476-4598-9-12 (PMC2823650; doi:10.1186/1476-4598-9-12)
Supplement: Additional file 1 — Supplementary Information. Supplementary methods, tables and figures. [file 1476-4598-9-12-S1.PDF]

Supplementary Information

miR-21: an oncomir on strike in prostate cancer

Marco Folini[1*], Paolo Gandellini[1*], Nicole Longoni[1], Valentina Profumo[1], Maurizio Callari[1], Marzia Pennati[1], Maurizio Colecchia[2], Rosanna Supino[1], Silvia Veneroni[1], Roberto Salvioni[3], Riccardo Valdagni[4], Maria Grazia Daidone[1], Nadia Zaffaroni[1§]

[1]Department of Experimental Oncology and Molecular Medicine, Fondazione IRCCS Istituto Nazionale dei Tumori, Via Venezian, 1, Milan, 20133, Italy; [2]Department of Pathology, Fondazione IRCCS Istituto Nazionale dei Tumori, Via Venezian, 1, Milan, 20133, Italy; [3]Department of Urology, Fondazione IRCCS Istituto Nazionale dei Tumori, Via Venezian, 1, Milan, 20133, Italy; [4]Prostate Program, Scientific Directorate, Fondazione IRCCS Istituto Nazionale dei Tumori, Via Venezian, 1, Milan, 20133, Italy

[*]These authors contributed equally to this work

[§]Corresponding author

SUPPLEMENTARY METHODS

Normal prostate cells and transfection conditions

Normal RWPE-1 prostate cells were obtained from American Type Culture Collection (Rockville, MD) and maintained in 5% CO_2 at 37°C in K-SFM culture medium (Invitrogen, Carlsbad, CA) supplemented with 5 ng/ml epidermal growth factor and 0.05 mg/ml bovine pituitary extract. Ectopic expression of miR-21 was pursued by exposing RWPE-1 cells to 20 nM miR-21 precursor (pre21) purchased as Pre-miR™ miRNA precursor molecule (Ambion, Austin, TX). Transfection was carried out using Lipofectamine2000™ (Invitrogen), according to the manufacturer's protocol. As a control for transfection experiments, cells were exposed to a negative control oligomer (preNeg, Ambion). The effect of ectopically expressed miR-21 on the growth of RWPE-1 cells was evaluated at day 3 after a 4-h transfection by counting cells in a particle counter (Coulter Counter, Coulter Electronics, Fullerton, CA).

RNase protection assay

The assay was performed using ^{32}P-labeled RNA probes obtained by *in vitro* transcription as described in Supplementary Table S1, using mirVana miRNA Probe Construction Kit (Ambion). The assay was carried out using mirVana miRNA Detection Kit (Ambion). Briefly, RNA samples (2 μg) were mixed with the specific probes, heat-denaturated and incubated at 42°C overnight to allow hybridization. Unhybridized RNA and excess probes were then removed by digesting with RNase A/RNase T1. The hybridized, protected RNA fragments were recovered by nucleic acid precipitation and electrophoresed on a denaturating polyacrylamide gel. Gel was then exposed to autoradiography films, and images acquired and elaborated using the EPSON Expression 1680 Pro scanner and Adobe Photoshop Image Reader 7.0, respectively.

Northern blotting

Total RNA was extracted from DU145 and PC-3 cells with Trizol Reagent (Invitrogen, Carlsbad, CA) according to the manufacturer's instructions. Total RNA (20 μg) was resolved on 15% polyacrylamide 7 mol/L urea gel and transferred to Hybond N+ membrane (GE Healthcare, Amersham, UK). An oligonucleotide probe complementary to the mature miR-21 (5'-TCAACAT CAGTCTGATAAGCTA-3') was 5'–labeled with [γ-^{32}P] adenosine triphosphate by T4 polynucleotide kinase (GE Healthcare). The membranes were incubated with ^{32}P-probe at 37°C for 16 h, in Rapid Hybridization Buffer (GE Healthcare). Membranes were washed at 42°C — twice with 2X SSPE (standard saline phosphate [0.18 mM NaCl/10 mM phosphate, pH 7.4], 1 mM EDTA) and twice with 0.5X SSPE/0.1% SDS — and subsequently visualized by autoradiography. Filters were stripped in boiling 0.1 % SDS for 10 min before rehybridization with ^{32}P-labeled U6 probe (5'-GCAGGGGCCATGCTAATCTTCTCTGTATCG-3'), used as loading control.

Luciferase reporter assay

For reporter assays, DU145 and PC-3 cells were transiently transfected with pGL3 basic vector (Promega, Madison, WI) or Luc-Pdcd4WT reporter plasmid, which contained the full-length 3'-UTR of Pdcd4 downstream of the luciferase gene (kindly provided by Dr. H. Allgayer, University of Heidelberg, Germany), and with miR-21 precursor (pre21) or antisense (LNA21) oligomers using Lipofectamine2000™ (Invitrogen). Reporter assay (Dual-luciferase-assay system, Promega) was performed 48 h post-transfection, according to the manufacturer's instructions. Normalization for transfection efficiency was performed by the cotransfection of *Renilla* luciferase. Scrambled precursor (preNeg) and antisense (LNAScr) oligomers were used as experimental controls.

Analysis of miR-21 expression data

MiR-21 expression levels in PCa and normal or benign samples were analyzed in publicly available (GSE8126, GSE2564, E-MEXP-1058) or kindly provided (Ozen *et al.*, 2008) normalized datasets. Using the R software, a two-sided Student's *t* test was computed and *P* values adjusted according to Bonferroni's correction for multiple tests.

**Dataset**	**Probe**	***t***	***P***	**Corrected *P***
Lu *et al.*, 2005 (GSE2564)	hsa-miR-21	-3.41	0.017	3.714
Porkka *et al.*, 2007 (E-MEXP-1058)	miR21	-3.28	0.047	9.781
Ozen *et al.*, 2008 (kindly provided)	hsa-miR-21	-3.18	0.0039	0.331
Ambs *et al.*, 2008 (GSE8126)	hsa-mir-21No2	1.07	0.293	183.435
	hsa-mir-21No1	-0.03	0.977	612.559

SUPPLEMENTARY TABLES

Table S1. Probes for RNase protection assay

DNA oligomer templates for *in vitro* transcription of RPA probes[1]	
miR-21	5'-TAGCTTATCAGACTGATGTTGATTTTCCTGTCTC-3'
5S RNA	5'-GATGGGAGACCGCCTCCTGTCTC-3'

[1]DNA oligonucleotide templates specific for the sequence to be detected and extended by an 8 base sequence complementary to the 3' end of a T7 promoter. By a fill-in reaction with Exo-Klenow, these oligomers were converted into double-stranded DNA templates ready for *in vitro* transcription with T7 RNA polymerase. After transcription in the presence of [α-^{32}P]-UTP, radiolabeled RNA probes were treated with DNase, to remove residue DNA template, and gel-purified. Only fragments corresponding to the full length probes were then excised and used in RPA experiments.

Table S2. Primers used for end-point RT-PCR

Primers designed using Primer3 online tool[1]

Gene	Forward	Reverse
PTEN	5'-GTTTACCGGCAGCATCAAAT-3'	5'-CCCCCACTTTAGTGCACAGT-3'
PDCD4	5'-TTTGGGTACAAGGCATTTCTG-3'	5'-GGAGTGGCAGTCAGACATCA-3'

[1]Steve Rozen and Helen J. Skaletsky (2000) Primer3 on the WWW for general users and for biologist programmers. In: Krawetz S, Misener S (eds) *Bioinformatics Methods and Protocols: Methods in Molecular Biology*. Humana Press, Totowa, NJ, pp 365-386.

Table S3. Commercial assays used for quantitative RT-PCR

Gene	Assay
miR-21	TaqMan® microRNA assay PN4373090 (Applied Biosystems)
U6	TaqMan® microRNA assay PN4373381 (Applied Biosystems)
PTEN	TaqMan® gene expression assay Hs02621230_s1 (Applied Biosystems)
RNP	TaqMan® RNaseP control reagent kit PN4316844 (Applied Biosystems)

Table S4. IC$_{50}$ values of Cisplatin and Taxol

	DU145		PC-3	
	Cisplatin (μM)	Taxol (nM)	Cisplatin (μM)	Taxol (nM)
Untreated	9.60±1.0	6.06±0.27	9.0±0.17	5.43±0.19
LNAScr	10.20±1.3	5.53±0.14	9.6±1.1	5.66±0.10
LNA21	9.22±0.57	5.50±0.10	10.1±2.0	5.43±0.10

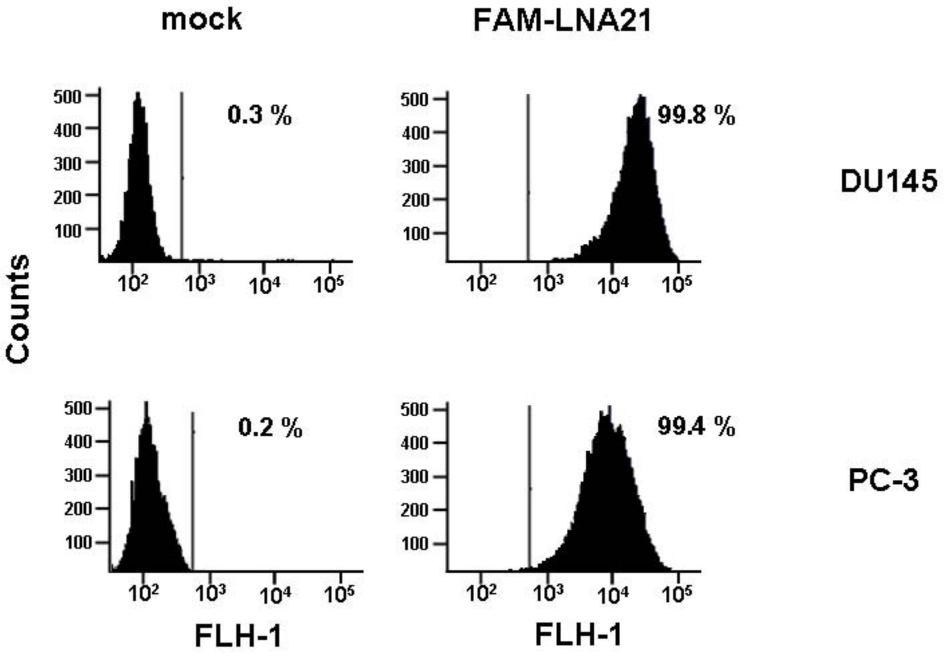

Figure S1. Evaluation of transfection efficiency in DU145 and PC-3 cells by flow cytometry. Cells seeded at the appropriate density were transfected for 4 h at 37°C with 100 nM FAM-LNA21 (carboxyfluorescein-labeled anti-miR-21 LNA oligomer, Exiqon, Vedbaek, Denmark) using Lipofectamine2000™ (Invitrogen), according to the manufacturer's instructions. Transfection efficiency was evaluated by flow cytometry 24 h after transfection. The percentage of transfected cells is reported. Mock: FAM-LNA21 without transfection reagent.

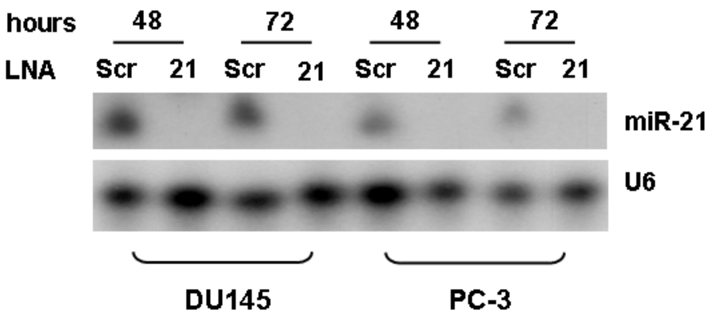

Figure S2. Evaluation of LNA-mediated depletion of miR-21 in PCa cells by northern blotting. Representative northern blotting showing the depletion of miR-21 in DU145 and PC-3 cells at 48 and 72 h after a 4-h transfection with LNA21, compared to LNAScr-treated cells. Lower panel shows the U6 RNA used as loading control.

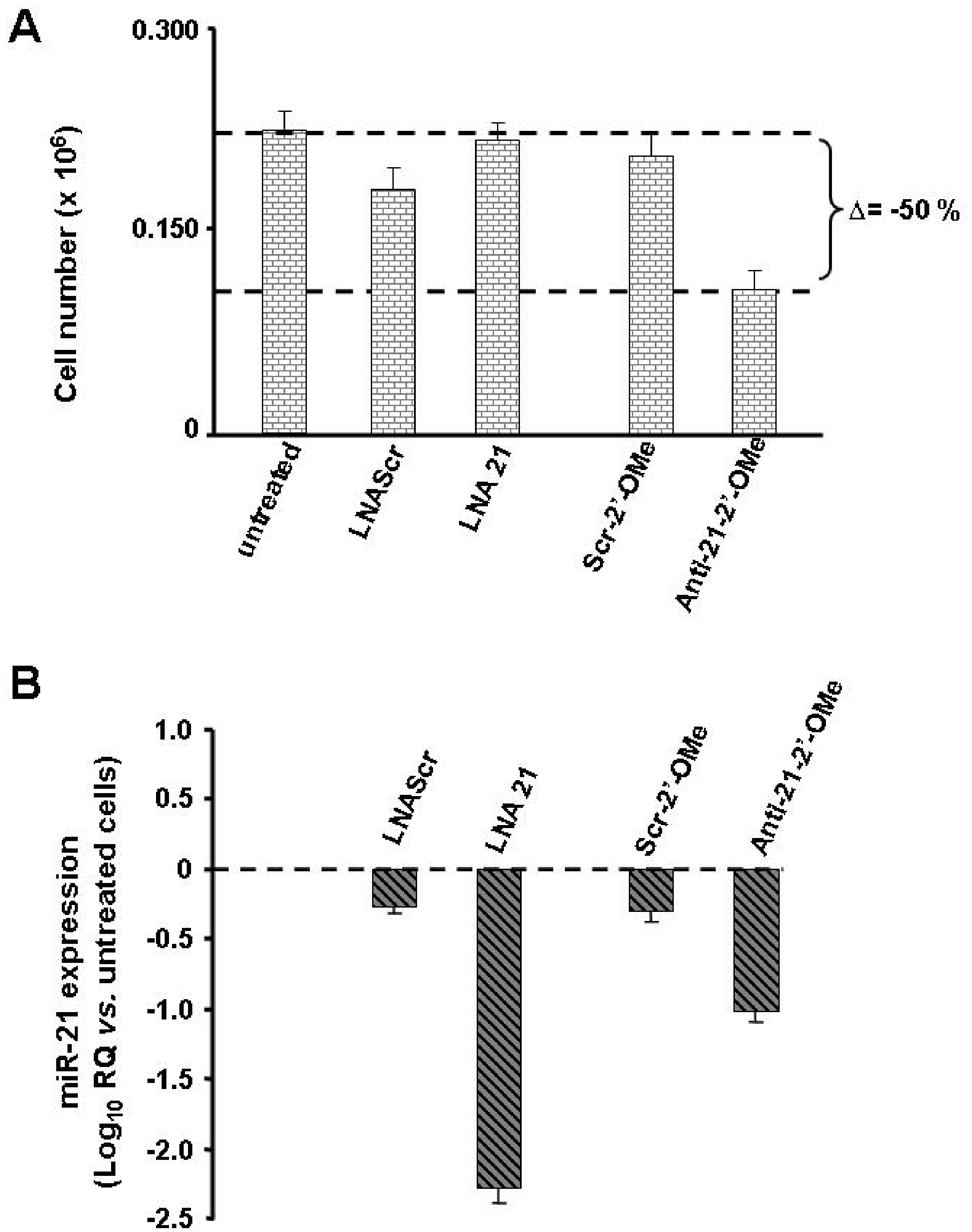

Figure S3. Effects of the different antisense-based approaches on cell growth and miR-21 expression in DU145 cells. (A) Bar graph showing the growth of DU145 cells, untreated or exposed to an equimolar amount of scrambled oligomers (LNAScr and Scr-2'-OMe) or anti-miR-21 oligonucleotides (LNA21 and Anti-21-2'-OMe). Data are reported as average cell number ± SD of

at least three independent experiments. (B) Quantification of miR-21 expression in DU145 cells exposed to the distinct antisense oligomers, assessed by qRT-PCR. Data are reported as Log_{10}-transformed relative quantity (RQ) of miR-21 expression in oligomer-treated cells with respect to untreated cells and represent mean values ± SD of at least three independent determinations. 2'-*O*-Methyl phosphorothioate oligomers (2'-OMe) were designed according to the sequence of the miR-21 mature form (miRBASE, http://microrna.sanger.ac.uk/) and chemically synthesized by Eurofins.

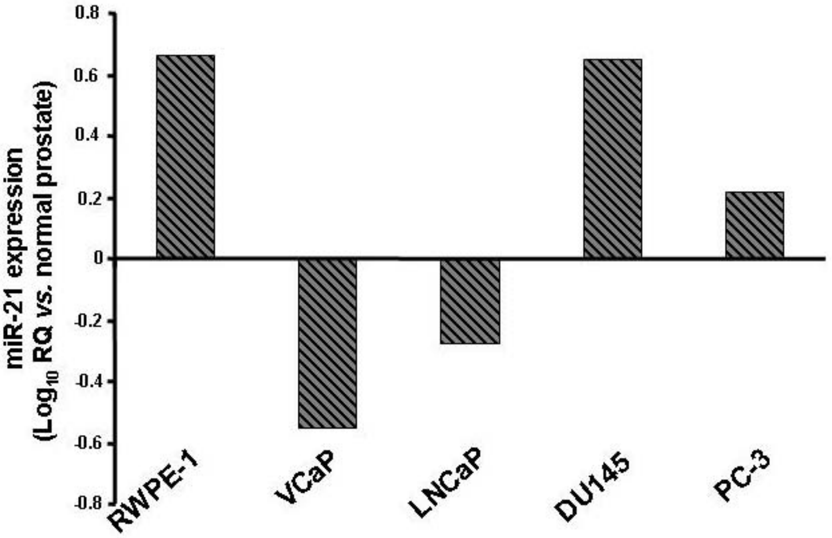

Figure S4. miR-21 was not differently expressed in tumor *vs.* normal prostate cell lines. The bar graph shows the quantification of miR-21 expression, assessed by qRT-PCR, on total RNA from normal RWPE-1 prostate cells, androgen-dependent (VCaP and LNCaP) and androgen-refractory (DU145 and PC-3) PCa cells, as previously obtained as described by Gandellini *et al.* (Gandellini *et al.*, 2009). Data are reported as Log_{10}-transformed RQ of miR-21 expression with respect to an internal calibrator (normal prostate RNA purchased from Ambion).

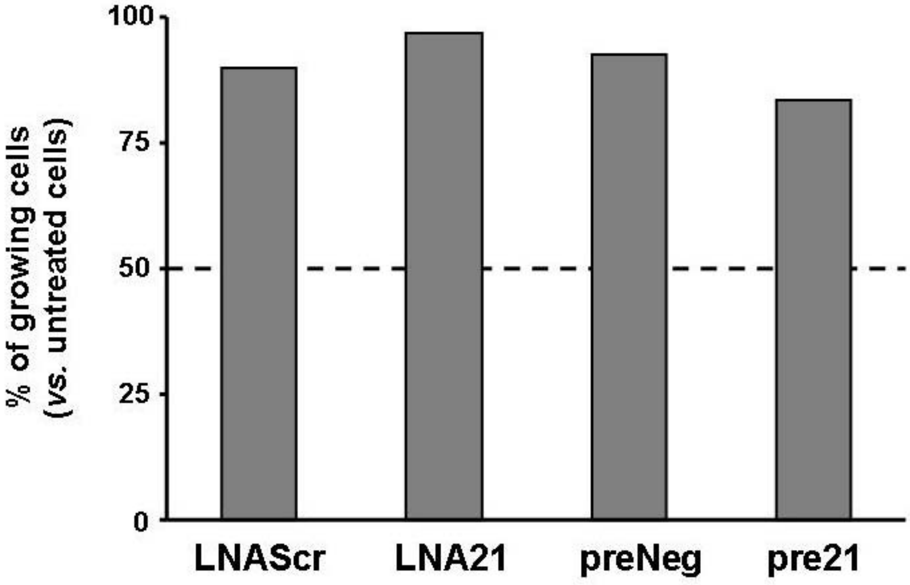

Figure S5. miR-21 down- or up-regulation failed to affect the growth of normal RWPE-1 prostate cells. The graph shows the percentage of growing RWPE-1 cells exposed to control oligomers (LNAScr and preNeg), anti-miR-21 LNA (LNA21) or synthetic miR-21 precursor oligomer (pre21), with respect to untreated cells. Data are reported as mean values of three independent determinations.

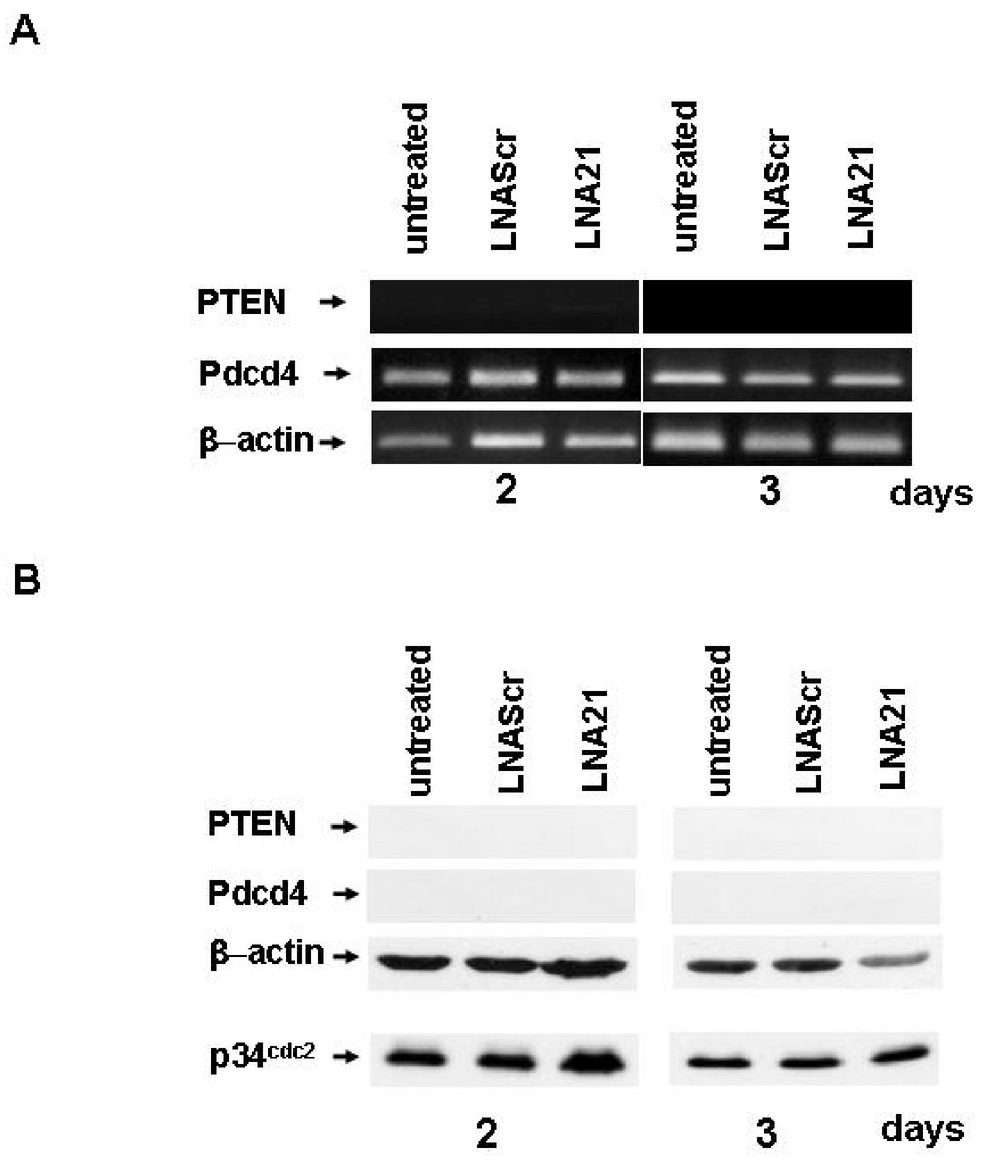

Figure S6. The depletion of miR-21 did not significantly affect the expression of its validated downstream targets. (A) RT-PCR showing the expression of known miR-21 targets in untreated or LNAScr− or LNA21−transfected PC-3 cells. β−actin was used as an internal PCR control. (B) Representative western immunoblotting showing the expression of miR-21 target proteins PTEN and Pdcd4, and its downstream effector $p34^{cdc2}$, in PC-3 cells at days 2 and 3 after exposure to LNAScr or LNA21. β−actin was used to ensure equal protein loading.

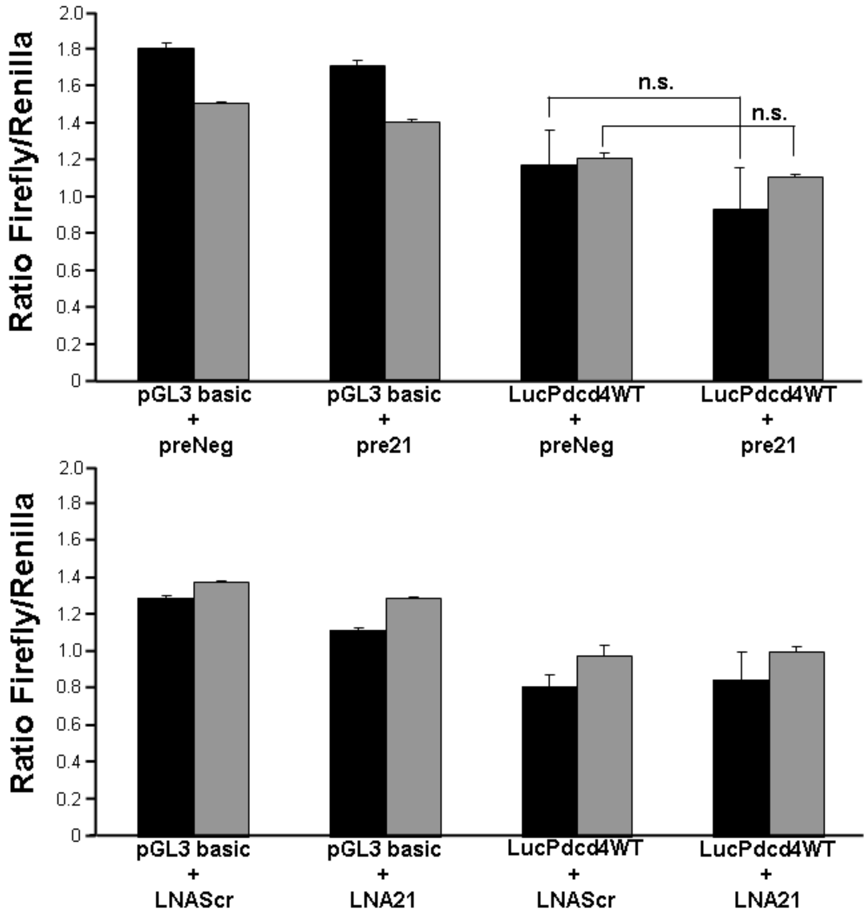

Figure S7. Luciferase reporter assay. Reporter assay in DU145 (black bars) and PC-3 (grey bars) cells co-transfected with pGL3 basic vector or Luc-Pdcd4WT reporter plasmid and miRNA precursor (preNeg and pre21) or antisense (LNAScr and LNA21) oligomers. Data are reported as ratio of firefly luciferase over *Renilla* luciferase luminescence signals. Data are reported as mean values ± SD from three independent experiments. n.s., not statistically significant.
